# Analysis of Hospitalization and Mortality by Race and Ethnicity Among Adults With Variceal Upper Gastrointestinal Hemorrhage, 2008-2018

**DOI:** 10.1001/jamanetworkopen.2022.22419

**Published:** 2022-07-18

**Authors:** Umer Farooq, Zahid Ijaz Tarar, Harleen Kaur Chela, Veysel Tahan, Ebubekir Daglilar

**Affiliations:** 1 Department of Internal Medicine, Loyola Medicine/MacNeal Hospital, Berwyn, Illinois; 2 Department of Internal Medicine, University of Missouri, Columbia; 3 Division of Gastroenterology and Hepatology, Department of Medicine, University of Missouri, Columbia

## Abstract

This cohort study examines mortality associated with variceal upper gastrointestinal hemorrhage during an 11-year period by race and ethnicity.

## Introduction

The overall mortality trends from variceal upper gastrointestinal hemorrhage (VUGIH) have been reported as decreasing (1989-1999) with an undulating incidence trend.^1^ There is limited literature addressing racial and ethnic differences in longitudinal incidence and related mortality trends of VUGIH.^2^ We conducted a longitudinal assessment of the racial and ethnic breakdown of incidence and mortality from VUGIH in the US during 11 years (2008-2018).

## Methods

This cohort study was a retrospective longitudinal trend analysis using National Inpatient Sample following the STROBE reporting guideline.^3^ All adults (age ≥18 years) with VUGIH were identified using the *International Classification of Diseases, Ninth Revision* (*ICD-9*) and *International Statistical Classification of Diseases and Related Health Problems, Tenth Revision* (*ICD-10*) codes for the corresponding years. Multilevel logistic mixed-effects regression models were used to address sample clustering (eMethods in the Supplement). The yearly incidence rate per 100 000 was calculated based on the US population estimate on July 1 of the corresponding year obtained from the US Census Bureau. Stata, version 14.2 (StataCorp LLC) was used to perform analyses, with 2-sided *P* < .05 considered statistically significant. The institutional review board of Loyola University Medical Center deemed the study exempt from review and the requirement for informed consent owing to the use of deidentified publicly available data.

## Results

A total of 234 982 patients with VUGIH were included. Mean age at presentation increased longitudinally, and hospital length of stay decreased from 6.45 days in 2008 to 6.17 days in 2018 (*P* = .01) (Table). Men had more hospitalizations than women during the entirety study (men, 26 615 [67.6%] to 26 223 [70.1%] vs women, 11 169 [29.9%] to 12 780 [32.4%]). The overall incidence of VUGIH increased from 11.82 per 100 000 persons in 2008 to 13.67 per 100 000 persons in 2018 (*P* < .001). Incidence trends increased from 2008 to 2018 in Hispanic and White individuals, whereas stable trends were noticed in Asian and Black individuals (Figure). The overall adjusted mortality from VUGIH decreased from 10.9% in 2008 to 9.6% in 2018 (*P* = .003). Mortality rates decreased in Black (*P* = .03 for trend) and Hispanic (*P* = .004 for trend) individuals only on longitudinal analysis. Racial and ethnic mortality comparison showed that, compared with White patients, Black patients had greater odds of mortality (adjusted odds ratio [aOR], 1.53; 95% CI, 1.06-2.21; *P* = .02), and Hispanic patients had lower mortality (aOR, 0.73; 95% CI, 0.54-0.98; *P* = .04). A higher proportion of rebleeding, fewer early (within 24 hours) endoscopy, and delayed presentation marked by advanced disease on hospital admission represented some of the reasons for higher mortality in Black patients.

**Table.** Characteristics of Patients Hospitalized With Variceal Upper Gastrointestinal Hemorrhage, 2008-2018

Variable	Hospitalizations by year, No. (%)						*P* value^a^
	2008	2010	2012	2014	2016	2018	
No. of hospitalizations	36 116	37 392	38 625	38 785	39 419	44 645	NA
Incidence per 100 000 persons	11.82	12.09	12.31	12.18	12.21	13.67	<.001
Sex							
Male	24 743 (68.5)	26 223 (70.1)	26 908 (70.0)	26 510 (68.4)	26 629 (67.6)	30 380 (68.0)	.18
Female	11 373 (31.5)	11 169 (29.9)	11 717 (30.3)	12 275 (31.6)	12 790 (32.4)	14 265 (32.0)	.18
Age, mean, y	55.99	55.40	56.03	56.48	56.44	56.59	<.001
Length of hospital stay, mean, d	6.45	6.45	6.33	6.29	6.15	6.17	.01
Mortality rate in different racial groups^b^							
Asian	58 (7.3)	118 (16.0)	70 (8.4)	130 (12.6)	140 (14.2)	85 (7.3)	.71
Black	395 (18.2)	482 (16.9)	435 (16.2)	440 (15.0)	525 (17.5)	455 (15.0)	.03
Hispanic	609 (10.7)	567 (8.1)	715 (9.4)	645 (8.4)	650 (8.4)	660 (7.3)	.004
White	2054 (10.6)	2118 (9.8)	2224 (9.5)	2265 (9.8)	2315 (9.7)	2640 (9.5)	.06
Overall	3935 (10.9)	3703 (10.0)	3895 (10.1)	3970 (10.2)	4020 (10.2)	4280 (9.6)	.003

^a^ Linear trend *P* value.

^b^ Adjusted for age and sex.

**Figure.**
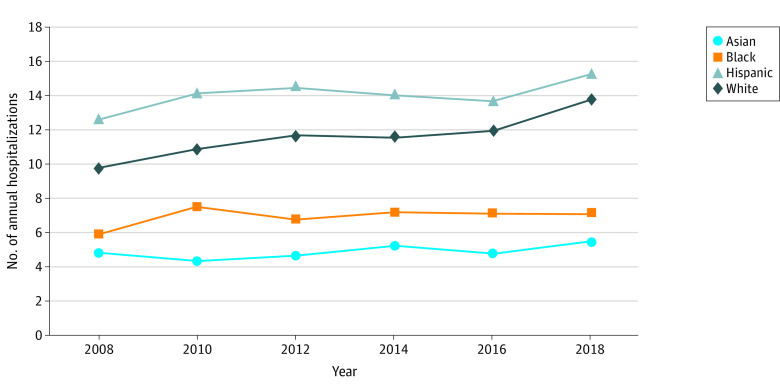
Trends in Racial and Ethnic Distribution of Patients Hospitalized With Variceal Upper Gastrointestinal Hemorrhage, 2008-2018

## Discussion

Hispanic individuals were found to have the highest incidence of VUGIH, followed by White individuals, with an increasing trend in the studied cohort. Contrary to the incidence, Black individuals had the highest mortality rate compared with White individuals, and Hispanic individuals had lower mortality rates. These racial and ethnic differences could occur due to differences in the prevalence of risk factors, including smoking, alcohol consumption, hepatitis, steatohepatitis, platelet inhibition, anticoagulant use, comorbidities, and delayed and disproportionate access to health care, as previous literature suggested^2^; however, further research may help elucidate whether racial and ethnic or genetic factors are also at play. In this cohort, overall mortality showed a downward longitudinal trend in all groups, likely partly due to readily available direct-acting antivirals for hepatitis C infection and its eradication.^4^ Despite this decreasing trend, Black individuals had 52% higher odds of mortality than White individuals. Nationwide strategies are needed to further reduce the mortality gap among different racial and ethnic groups. Nonalcoholic steatohepatitis is projected to be the leading cause of liver disease–related mortality and morbidity; several clinical trials testing various drugs are currently under development.^5^ A limitation of this study is the inability to control for cirrhosis stage based on Child-Pugh or Model for End-stage Liver Disease score and reliance on *ICD* codes. However, we applied the Charlson Comorbidity Index for comorbidity burden and also controlled for major comorbidities included in the Rockall score.

## Notes

Supplement. **eMethods.** Additional Methods
