# Supplementary material for: Analysis of Hospitalization and Mortality by Race and Ethnicity Among Adults With Variceal Upper Gastrointestinal Hemorrhage, 2008-2018
Source: JAMA Netw Open. 2022 Jul 18;5(7):e2222419. doi: 10.1001/jamanetworkopen.2022.22419 (PMC9294996; doi:10.1001/jamanetworkopen.2022.22419)
Supplement: Supplement. — eMethods. Additional Methods [file jamanetwopen-e2222419-s001.pdf]

Supplementary Online Content

Farooq U, Tarar ZI, Chela HK, Tahan V, Daglilar E. Analysis of hospitalization and mortality by race and ethnicity among adults with variceal upper gastrointestinal hemorrhage, 2008-2018. *JAMA Netw Open*. 2022;5(7):e2222419.
doi:10.1001/jamanetworkopen.2022.22419

eMethods. Additional Methods

This supplementary material has been provided by the authors to give readers additional information about their work.

© 2022 Farooq U et al. *JAMA Network Open.*

eMethods. Additional Methods

We conducted a retrospective longitudinal trend survey using National Inpatient Sample (NIS) following STROBE guidelines. It is designed as a stratified sample of 20% of all hospital stays in the USA. Each hospital discharge is then weighted (weight=total number of discharges from all acute care hospitals in the USA divided by the number of discharges included in the 20% sample), making it nationally representative. Using the International Classification of Diseases (ICD), Ninth Revision, and ICD, Tenth Revision codes for the corresponding years, we included adult patients (≥18 years old) with variceal upper gastrointestinal hemorrhage. The control population consisted of all adult hospitalizations. Race variable is available within NIS and contains a uniform coding for race and ethnicity. If the data source supplied information on race and ethnicity as separate data elements, ethnicity took precedence over race in setting the uniform values for the variable. We followed Healthcare Cost and Utilization Project recommendations to use revised trend weights to obtain proportionate estimates. Multilevel mixed-effects regression models were used to address sample clustering. Other variables adjusted for in the regression models were: gender, age, Charlson Comorbidity Index score, median household income for patients' zip codes, hospital location/region/ bedside, admission on the weekend, and teaching status. We also added major comorbidities included in the Rockall score in the regression model (including heart failure, ischemic heart disease, renal failure, liver failure, metastatic cancer, as well as the presence of shock). For the other calculations, proportions were compared using the Fisher exact test, and continuous variables were compared using the Student's t-test. The yearly hospitalization rate per 100,000 was calculated by the US population estimate on July 1 of the corresponding year obtained from the US Census Bureau. We used Stata, version 14.2, to perform analyses considering 2-sided $P < .05$ as statistically

© 2022 Farooq U et al. *JAMA Network Open.*

significant. The Institutional Review Board approval was sought, but the study was deemed exempt, and informed consent was waived due to the use of de-identified publicly available data.

© 2022 Farooq U et al. *JAMA Network Open.*
